# Atrioventricular Nodal Reentrant Tachycardia Ablation with a Power-controlled, Contact-force Catheter

**DOI:** 10.19102/icrm.2020.111103

**Published:** 2020-11-15

**Authors:** Chase J. Contino, Max Weiss, Michael P. Riley, Daniel R. Frisch

**Affiliations:** ^1^Department of Cardiology, Thomas Jefferson University, Philadelphia, PA, USA; ^2^Department of Cardiology, Hospital of the University of Pennsylvania, Philadelphia, PA, USA

**Keywords:** Atrioventricular nodal reentrant tachycardia, electrogram, radiofrequency catheter ablation

## Abstract

Radiofrequency catheter ablation is a safe and effective treatment option for atrioventricular nodal reentrant tachycardia (AVNRT). A nonirrigated ablation catheter used in a temperature-controlled mode is traditionally used for AVNRT ablation due to the shallow lesion depth required for successful slow-pathway ablation. In this case, a nonirrigated ablation catheter established inadequate lesions to ablate the slow pathway successfully. The adoption of an irrigated contact-force ablation catheter used in a power-controlled mode was necessary to provide higher power and possibly create a deeper lesion to ablate the slow pathway successfully, thus eliminating AVNRT inducibility in this patient.

## Introduction

Radiofrequency catheter ablation (RFA) is a safe and effective treatment option for atrioventricular nodal reentrant tachycardia (AVNRT). A nonirrigated ablation catheter used in a temperature-controlled mode is traditionally adopted for AVNRT ablation due to the shallow lesion depth required for successful slow-pathway ablation. However, energy delivery is limited due to the temperature at the catheter–tissue interface. Irrigated ablation catheters are not limited by the catheter–tissue interface temperature and are capable of greater energy delivery, creating larger and deeper lesions.^1^

## Case description

**Figure 1:**
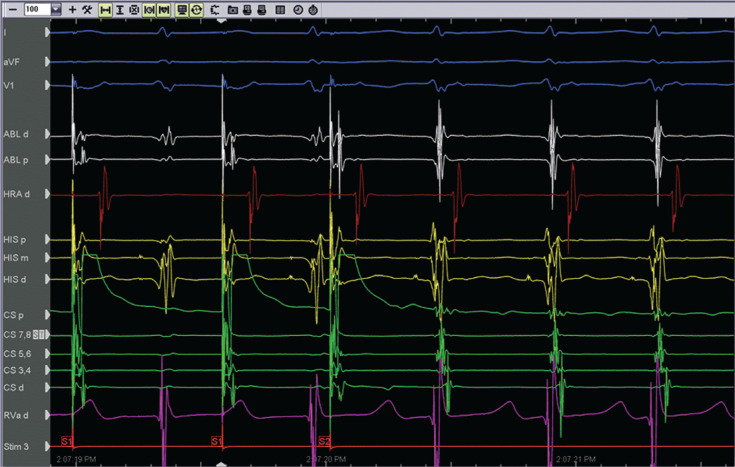
Atrial extra-stimulus causing an AH “jump” that initiated a narrow-complex, short R–P, A-on-V tachycardia consistent with AVNRT.

**Figure 2:**
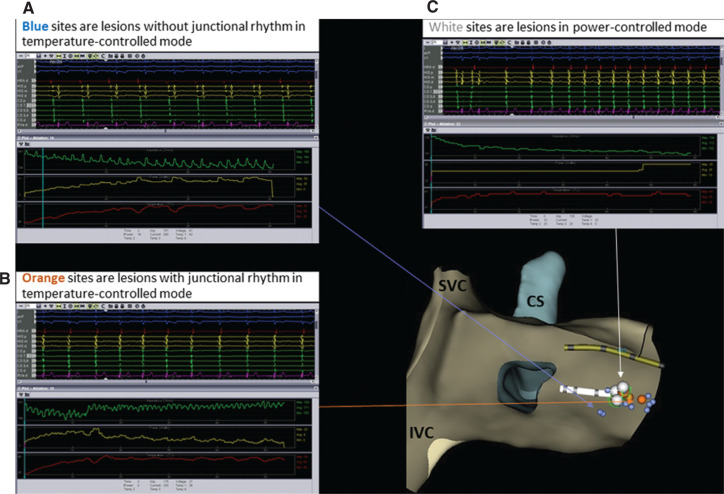
Ablation sites. The green, yellow, and red lines represent impedance, power, and temperature, respectively. **A:** Blue markers: temperature-controlled mode without junctional rhythm. **B:** Orange markers: temperature-controlled mode with junctional rhythm. **C:** White markers: power-controlled mode with junctional rhythm. CS: coronary sinus; IVC: inferior vena cava; SVC: superior vena cava.

A 73-year-old man with a history of persistent atrial fibrillation who had undergone pulmonary vein isolation 29 months earlier presented with palpitations. His electrocardiogram revealed a narrow complex, short R–P tachycardia consistent with AVNRT and he was referred to electrophysiology for RFA. The electrogram recorded during supraventricular tachycardia (SVT) induction can be seen in **Figure 1**. The SVT was initiated with an atrio–His (AH) “jump” and shows a narrow-complex, short R–P, A-on-V tachycardia consistent with AVNRT. RFA of the slow-pathway region was initially attempted using a 4-mm, large, curved, nonirrigated ablation catheter (Blazer; Boston Scientific, Natick, MA, USA) set in the temperature-controlled mode. Initially, adequate temperatures were achieved with adequate power delivery (50 W and a temperature of 50°C–65°C). However, the desired ablation effect, an accelerated junctional rhythm, was not observed **(Figure 2A)**.

With adjustment of the catheter position, a junctional rhythm was observed with ablation; however, the average power delivery was consistently less than 10 W despite a temperature cutoff of 65°C **(Figure 2B)**.

Despite the creation of a total of 20 lesions, AVNRT was still able to be induced. The use of both a long sheath (SR0 curve) and a steerable sheath (Agilis medium curl; Abbott Laboratories, Chicago, IL, USA) did not improve results. The induction of junctional rhythm with ablation using the nonirrigated catheter indicated that the catheter position was in the appropriate location for slow-pathway modification of the atrioventricular (AV) node. It was noted that the power delivery was low during the creation of these lesions and it was decided that a deeper lesion might instead suffice and could be more durable. Therefore, no attempt was made to ablate in the proximal coronary sinus using the nonirrigated catheter.

To deliver more power to the slow pathway, the 4-mm nonirrigated catheter was removed and a 3.5-mm irrigated contact-force catheter (75 Tacticath; Abbott Laboratories, Chicago, IL, USA) was placed. Power was titrated between 20 W and 25 W and the flow was adjusted from the usual 17 mL/min down to the lowest programmable setting of 6 mL/min during ablation. Two new lesions were delivered in the same area where the junctional rhythm was previously induced, with successful induction of the junctional rhythm. These lesions are represented by white markers in **Figure 2**. The ablation characteristics of the lesion set are summarized in **Table 1**.

At the conclusion of these lesions, AVNRT was no longer inducible, even with the infusion of isoproterenol, and an AV-node effective R–P interval was observed for the first time (600/470 ms). Ultimately, AV nodal Wenckebach occurred at 400 ms and the ventricular effective R–P interval was 600/210 ms. Therefore, it was decided that no further ablation was required.

The patient tolerated the procedure without any significant complications and was followed for 39 months postablation. There was no evidence of SVT on the patient’s personal electrocardiogram monitor (KardiaMobile; AliveCor, Mountain View, CA, USA) and no reported symptoms.

## Discussion

This case describes a unique solution during a challenging AVNRT ablation procedure. Initial attempts at slow-pathway region ablation for typical AVNRT were unsuccessful using a nonirrigated catheter in a temperature-controlled mode; power delivery was inadequate, with temperatures approaching a cutoff as high as 65°C. Other investigators have conducted a retrospective cohort analysis of all ablation modalities when ablating AVNRT, including nonirrigated and irrigated RFA and cryoablation, and found that the majority of operators use nonirrigated catheters; however, the use of irrigated contact-force catheters has slowly been increasing over time. They also found that, regardless of ablation modality, AVNRT ablation was safe and highly effective.^2^ Elsewhere, researchers used a flexible-tip irrigated catheter for AVNRT ablation and similarly reported high levels of safety and efficacy.^3^ A study of the biophysics at the catheter electrode–endocardial interface showed that saline-irrigated radiofrequency applications resulted in larger lesions by delaying or eliminating the impedance rise, despite the presence of electrode–endocardial interface boiling. Electrode coagulum was consistently associated with impedance rise, which was delayed or eliminated with irrigation. They also found that a higher irrigation rate was associated with the elimination of electrode coagulum and impedance rise. The maximum size of an irrigated lesion was well beneath the endocardial surface, as opposed to nonirrigated ablation, which exhibited the maximum size at or very close to the endocardial surface.^4^

For this case, it was hypothesized that the lesion depth was inadequate. The strategy to use an irrigated contact-force ablation catheter with the lowest programmable irrigation flow rate was designed to increase the depth of the ablation lesion without increasing the lesion size excessively, which would risk damage to the compact AV node. The intention was to deliver more power to the slow-pathway region and create a more durable lesion.

Although temperature monitoring during ablation with an irrigated catheter is limited relative to a nonirrigated catheter, the use of an irrigated catheter was necessary to deliver higher power and possibly a deeper lesion to successfully ablate the slow-pathway region in this case. There was no direct requirement for the involvement of contact-force technology in this case. However, the chosen ablation catheter incorporating a power-controlled mode also included contact-force sensing. Therefore, the contact force was monitored to confirm there was adequate catheter–endocardial contact that would allow the application of more durable lesions.

## Figures and Tables

**Table 1:** Ablation Characteristics of the Two Lesions that Successfully Ablated the Slow-pathway Region

Lesion	FTI (LSI)	Average/Max Force	Power	Impedance Drop
1 (60 seconds)	864 (5.2)	14/28 g	20–25 W at 48 seconds	From 139 to 118 Ω = 21 Ω
2 (60 seconds)	442 (4.6)	7/11 g	20–25 W	From 144 to 109 Ω = 35 Ω

FTI: force–time integral; LSI: lesion size index.
